# Contributions of Dietary Patterns and Factors to Regulation of Rheumatoid Disease

**DOI:** 10.3390/ijms26062674

**Published:** 2025-03-16

**Authors:** Jingjie Zhang, Xueli Wang, Juan Fang, Yingying Li, Yonghui Yu, Jing Wang, Baoguo Sun

**Affiliations:** 1 Key Laboratory of Geriatric Nutrition and Health, Ministry of Education, Beijing Technology and Business University, Beijing 100048, China; jingjiezhang@btbu.edu.cn (J.Z.); wangxueli521856@163.com (X.W.); fangjuan991129@163.com (J.F.); liyingying_siren@163.com (Y.L.); sunbg@btbu.edu.cn (B.S.); 2 China-Canada Joint Laboratory of Food Nutrition and Health, Beijing Technology and Business University, Beijing 100048, China; 3 Key Laboratory of Special Food Supervision Technology for State Market Regulation, Beijing Technology and Business University, Beijing 100048, China

**Keywords:** rheumatoid arthritis, dietary pattern, risk factors, mitigating factors

## Abstract

Rheumatoid arthritis (RA) is a systemic autoimmune disease that commonly causes pain in joints and the progressive destruction of cartilage and bone, which significantly reduces the quality of life and increases the social burden. However, there is still no cure for RA, so it is highly important to explore additional adjuvant treatment methods. Studies have indicated that malnutrition, changes in intestinal microbiota, and changes in immune status caused by dietary imbalance are directly related to the onset of RA, indicating that dietary intervention may offer a simple, economical, and practical avenue to relieve RA. Therefore, in this review, we discuss the pathogenesis of RA and summarize the influence of different dietary patterns on RA. In particular, we pointed out that high-fat, high-sugar, and high-salt diets contribute to RA progression, whereas the Mediterranean diet (MD) is beneficial for preventing RA. Furthermore, the ingredients of food, such as dietary fiber, probiotics, and vitamins, help reduce the level of inflammation and relieve joint pain, which may play critical roles in the treatment of RA. Therefore, dietary intervention provides a potential effective approach for adjuvant therapy of RA.

## 1. Introduction

RA is a chronic systemic autoimmune disease, characterized by painful and swollen joints, leading to irreversible joint deformity, which can significantly impair physical function [1]. RA was first considered a chronic disorder that causes synovial inflammation and proliferation, autoantibody production, and destruction of cartilage and bone [2]; however, further studies have shown that RA can also impair a wide variety of body systems, such as the lung, kidney, liver, and even cardiovascular systems [3], both of which severely reduce quality of life [4]. Although the prevalence of RA varies globally, the number of RA patients worldwide has been rising annually. From 1990 to 2017, the global age-standardized RA incidence rate increases by 7.4% [5]. The situation is similar in China, where the incidence rate of RA has reached 0.42% on the basis of the latest statistical data [5,6]. According to the predicted population changes, it is estimated that there will be 31.7 million patients with RA worldwide in 2050, which will increase the number of cases by 80.2% from 2020 to 2050 [6], severely increasing the social and medical burden.

**Figure 1:**
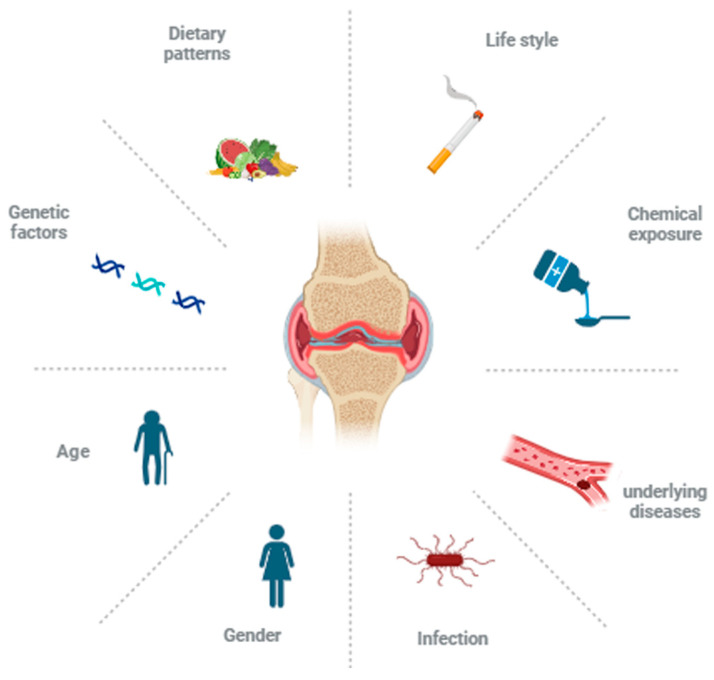
The induced factors for RA. The risk factors for RA are divided into genetic and non-genetic factors. *HLA* genes are strongest correlation with RA occurrence. Non-genetic factors include age, gender, infection, chemical exposure, basic disease, life style, and dietary pattern.

Numerous studies have shown that genetics, the environment, immunity, and other factors play key roles in the pathogenesis of RA [7] (Figure 1). Genetic factors are mainly divided into human leucocyte antigen (*HLA*) genes and other genes, and some alleles of the *HLA* genes are closely related to the susceptibility to RA [8]. For example, the *HLA-DRB1* gene, such as HLA-DRB1*0401, HLA-DRB1*0404, and HLA-DRB1*0101, has been shown to be the genetic factor with the strongest correlation with RA [9]. These genes may change the process of antigen presentation, and the immune system will wrongly recognize and attack joint tissues [10]. In addition, polymorphisms in *PTPN22*, *CTLA-4,* and other genes have also been considered to be associated with RA [11]. Therefore, although RA is not directly heritable, genetics and family history have been found to be relevant to the increasing risk of RA [12].

However, genetic predisposition accounts for only 30–60% of the risk of RA, whereas 40–70% of RA risk is influenced by non-genetic factors [13]. As shown in Figure 1, the onset age of RA is usually between 30 and 50 years, reaching its peak between 50 and 75 years, and approximately 75% of patients are women [14], indicating that age and sex also have a significant effect on the risk of RA. Non-genetic factors, including the following aspects, have an important influence on the onset of RA [15]: (1) Infection: Microbial infection is thought to play an important role in the occurrence and persistence of RA. The results of clinical trials and animal model studies have shown that multiple microbial infections, including *Porphyromonas gingivalis* [16], *Proteus mirabilis* [17], Epstein–Barr virus [18], and mycoplasma [19], may cause the onset of RA in susceptible individuals. (2) Chemical exposure: Long-term exposure to certain chemical substances (such as silica dust, organic solvents, and chemical components in tobacco, etc.) may increase the risk of RA [20]. (3) Underlying diseases: There is a close relationship between cardiovascular disease and RA. Research shows that patients with RA are at increased risk of cardiovascular disease [21], which has been at least partially attributed to elevated levels of inflammatory factors in the body. Research has confirmed this relationship from another perspective, that C-reactive protein (CRP) has shown an increasing trend in both of these diseases [22]. In addition, obesity is considered an important factor leading to an increase in systemic inflammatory levels and further increases the incidence and severity of RA [23]. Specifically, excessive body mass index may have negative impacts on patients with RA, including increased disease risk and disease activity index, reduced quality of life, and insensitivity to treatment [24]. (4) Lifestyle: A lifestyle of nonsmoking, healthy eating, and moderate exercise can increase the body’s antioxidant capacity [25], reduce inflammation [26], regulate immunity [27], improve joint function, and relieve pain [28], thereby lowering the incidence of RA. In contrast, lifestyle factors such as unhealthy diets, a lack of exercise, and/or smoking contribute to aggravated inflammation [29,30], reduced muscle mass [31], and increased joint stiffness [32], all of which may worsen symptoms of RA or increase the risk of developing RA. (5) Stress: Stress can not only affect the function of the immune system but also lead to an increase in the inflammatory response, thus aggravating the symptoms of rheumatoid arthritis. Stress can also trigger changes in hormone levels in the body, such as cortisol secretion, which may affect the body’s inflammatory response. (6) Other factors: Citrullination, a type of posttranslational modification, converts arginine to citrulline in certain proteins. Abnormal citrullination might disrupt the natural self-immune tolerance. Citrullinated proteins can be recognized as foreign antigens and trigger an immune response to produce anti-citrullinated autoantibodies, resulting in RA [32]. In addition, sex differences in immune responses are also important factors in triggering RA. This is why the incidence of RA in women is significantly greater than that in men [33].

Among these non-genetic factors, dietary factors, in particular, have been found to play critical roles in RA progression (Figure 1). Healthy dietary patterns, such as the Mediterranean diet (MD), which is rich in plant foods, whole grains, healthy fats, and fish, have been proven to be beneficial for reducing the risk of RA [34], whereas unhealthy dietary patterns, including high-sugar and high-fat diets, have been found to promote RA progression [35]. The fact that the impact of different dietary patterns on RA varies greatly is largely attributed to differences in the types and amounts of nutrients they contain [36]. The content and proportion of nutrients play important roles in the occurrence and severity of diseases. Studies have shown that some dietary choices may have pro-inflammatory or anti-inflammatory effects, thus affecting the immune system function in vivo [37].

Further studies have shown that some ingredients enriched in healthy diets have significant effects on reducing inflammatory levels in the body, which may contribute to the management of RA. For example, omega-3 fatty acids possess functions of immunomodulatory properties, anti-inflammatory reaction, and balancing gut microbiota and have been proven to be effective in relieving RA symptoms [38,39,40]. Clinical studies indicated that consumption of omega-3 fatty acids could significantly improve eight RA-related markers [41]. Vitamins and minerals are also essential for the normal function of the immune system. Deficiencies in vitamins D, C, and zinc may lead to immune system disorders, thus increasing the risk of RA [42]. As an antioxidant, vitamin E helps reduce oxidative stress and thus reduces the probability of RA [43]. Dietary fiber is another nutrient that is considered beneficial for RA management. On the one hand, dietary fiber can directly reduce systemic inflammation by enhancing intestinal barrier function [44]. On the other hand, dietary fiber contributes to improving the intestinal microbiota, and its metabolites, such as short-chain fatty acids, can regulate metabolism and immune status (promotion of regulatory T cells), thus indirectly alleviating RA [45]. Therefore, it is important to pay attention to diet structure and composition, especially for the management and prevention of RA. In this review, we summarize the influence of different patterns on RA progression and analyze the effects of nutrients enriched in healthy diets on relieving RA to provide a potential effective approach for adjuvant therapy of RA.

## 2. The Pathogenesis of RA

**Figure 2:**
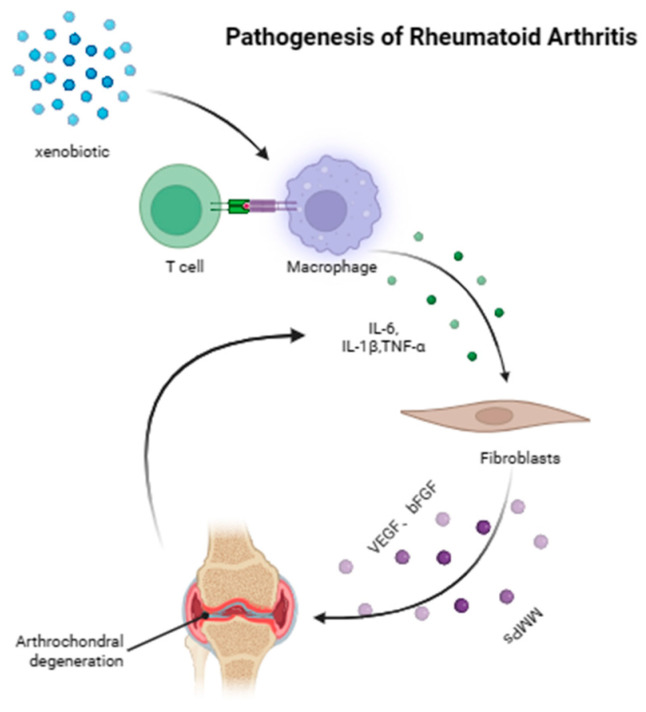
Pathogenesis of RA: First, the self-tissue is mistakenly identified as foreign substance, which leads to the activation of the immune response and the persistence of inflammation. Second, many cytokines are secreted and released. The proliferation and migration of synovial cells release proangiogenic factors, promote angiogenesis, and lead to joint swelling. Finally, persistent inflammation leads to articular cartilage degeneration and bone destruction, and inflammatory factors promote the secretion of MMPs, causing destructive damage to cartilage.

Although the pathogenesis of RA is not fully understood, it is generally believed that the development of RA includes the following stages (Figure 2): (1) The immune system mistakenly identifies self-tissues as heterologous substances, which leads to the activation of the immune response and the persistence of inflammation [46]. (2) Large amounts of cytokines are secreted and released, followed by increased immune cell and cytokine levels, especially in the joint tissue, which further promotes the inflammatory reaction [47]. (3) Inflammation leads to synovial cell proliferation and migration, leading to synovitis unique to RA [48], followed by the release of proangiogenic factors, such as vascular endothelia growth factor (VEGF) and basic fibroblast growth factor (bFGF), to promote angiogenesis [49]. In addition, inflammatory factors further increase vascular permeability, resulting in joint swelling [47]. (4) Continuous inflammatory reactions lead to the degradation of articular cartilage and bone destruction [50]. Large amounts of cytokines, such as interleukin-1β (IL-1β) and tumor necrosis factor-α (TNF-α), which are released by immune and synovial cells, promote the secretion of metalloproteinases (MMPs) [51]. MMPs can subsequently degrade cartilage matrix components, such as collagen fibers and proteoglycans, resulting in destructive damage to cartilage [52]. These inflammatory factors also directly stimulate the differentiation and activation of osteoclasts and promote bone resorption [53]. The destruction of cartilage and bone further stimulates synovial cells to secrete more inflammatory factors, forming a vicious cycle [54]. Therefore, inflammation serves as a critical stage in the pathogenesis of RA, and the inhibition of inflammation is considered a potentially effective way to relieve RA.

## 3. Effects of Different Dietary Patterns on RA Progress

The types and quantities of foods that people consume vary with region, habits, and personal preferences [55], and there are also significant differences in nutrient composition among these patterns [56]. Dietary patterns can be divided into balanced dietary patterns and unbalanced dietary patterns on the basis of whether they contribute to keeping us healthy, both physically and mentally [57]. The balanced diet pattern usually contains a variety of foods, which can provide enough macro- and micro-nutrients to meet the nutritional needs of the body [58], whereas the unbalanced dietary pattern lacks some nutrients, leading to malnutrition or related health problems [59]. As shown in Table 1, studies have shown that dietary patterns have a potentially important influence on the occurrence and development of RA [60].

### 3.1. Unbalanced Diet Patterns

#### 3.1.1. High-Fat Diet

A high-fat diet refers to a diet consisting of at least 35% of the total calories consumed from fats, including both unsaturated and saturated fats [73]. This diet pattern mainly contains foods enriched with animal fat (such as red meat, pork, full-fat dairy products, and poultry) and those rich in plant fat (such as vegetable oil, nuts, and seeds) [74], which usually results in high energy density. Common processing methods, such as frying, even increase the fat content.

**Figure 3:**
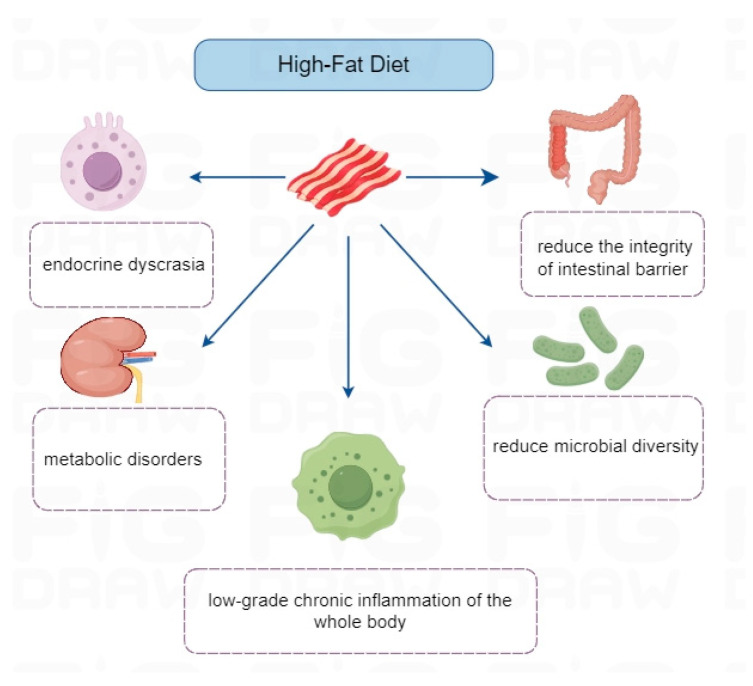
The potential harmful factors of a high-fat diet triggering RA: A long-term high-fat diet might result in endocrine dyscrasia, metabolic disorders, low-grade chronic inflammation of the whole body, reducing microbial diversity and the integrity of intestinal barrier, which contributes to inducing or aggravating RA.

The long-term consumption of high-fat diets not only leads to elevated blood lipids and obesity but also may result in a range of health complications, such as cardiovascular disease, metabolic syndrome, or gastrointestinal issues [61,62]. A previous study revealed that excess dietary intake disturbs the “clock” of the body, leading to metabolic disorders such as diabetes and obesity [75]. High-fat food is also considered one of the key factors of obesity and is often accompanied by a series of chronic inflammatory diseases, such as type II diabetes mellitus and cardiovascular diseases [76]. In addition, a high-fat diet contributes to low-grade chronic inflammation, endocrine dyscrasia, and metabolic disorders by damaging the integrity of the intestinal barrier and lowering microbial diversity, subsequently resulting in an imbalance of the immune system, which has been listed as one of the risk factors for RA (Figure 3). The result of clinical trial has shown that the symptoms of RA in patients with a high-fat diet is significantly more serious than those with an ordinary diet [77], and drugs against TNF-α are less effective in treating obese patients with RA [78], indicating the harmful effects of a high-fat diet on our health.

The effects of a high-fat diet on the risk of RA are multifaceted. First, excess fat intake increases the levels of pro-inflammatory factors, leading to an in vivo inflammatory environment. In a study with a collagen-induced RA animal model, mice fed a high-fat diet had significantly greater arthritis severity scores and a higher incidence of arthritis than C57BL/6 mice in control group at all time points and exhibited synovial pannus formation and progressive joint destruction [79]. Further results showed that the serum level of anti-IgG was elevated, and both the Th17 cell number and the mRNA level of *IL-17* in spleen cells were increased [79], suggesting that a high-fat diet may accelerate the inflammatory process and aggravate the symptoms of RA. Another study showed that a high-fat diet accelerated collagen-induced joint inflammation and RA occurrence in mice compared with a normal diet [80]. Subsequent results indicated that a high-fat diet stimulated the migration of neutrophils and led to the early onset of collagen-induced RA [80], suggesting that a high-fat diet may increase the infiltration of joint monocytes and accelerate the process of collagen-induced arthritis.

Other studies had shown that an increase in serum cholesterol is also related to the risk of RA. In particular, higher total cholesterol in women is positively correlated with increased long-term RA risk, which does not exist in men [81]. This trend showed that as a risk factor for RA occurrence, a high-fat diet may be more sensitive in women than in men [82]. In addition, sex-specific exposure alters the metabolism of lipids, and hormone-related metabolic pathways may also influence the occurrence of RA [81]. Taken together, a high-fat diet might promote the inflammatory reaction and further increase the risk of RA.

#### 3.1.2. High-Sugar Diet

Sugars include the sum of all the mono- and di-saccharides, such as glucose, fructose, and sucrose, and a high-sugar diet mainly refers to the daily intake of sugar exceeding 10% of the total energy [83,84]. High-sugar diets can be further divided into high-glucose diets and high-fructose diets [83]. According to the recommendation of the American Diabetes Association, the daily sugar intake of adults should be controlled between 25 g and 36 g [85]. The main sources of a high-sugar diet include sugary foods and drinks, such as candy, cakes, ice cream, and sugary soda water [86]. These foods have high sugar and calories content but low nutritional density and lack of dietary fiber, vitamins, and minerals [87]. Many studies have confirmed that long-term intake of a high-sugar diet may lead to metabolic diseases, such as obesity [88], insulin resistance [86], and fatty liver [89], and further increase the risk of cardiovascular disease and diabetes mellitus [90].

A high-sugar diet also contributes to an increased risk of RA. Clinical trials have shown that drinking sugary soda for a long period of time and the risk of RA onset are positively correlated, especially among women with a family history or other risk factors [91]. Animal studies had shown similar results that high-sugar diet intake induced more severe arthritis symptoms, including obvious joint swelling and tissue damage in a collagen-induced mouse model in comparison to those in a control group [63]. Further study found that high glucose intake increased blood sugar and insulin levels, activated inflammatory cytokines and chemokines, and thus aggravated the inflammatory reaction in individuals with RA [92]. A high-sugar diet led to significant inflammation and cell infiltration of the synovium, suggesting that sugar intake aggravated the inflammatory reaction in a collagen-induced mice model [63]. Other animal experiments also revealed that a high-sugar diet increased the levels of the inflammatory factors TNF-α and IL-6 in mice with RA, thus promoting the formation of osteolytic cells [64]. In addition, a high-sugar diet affects RA via various other pathways. A high-sugar diet increases the accumulation of advanced glycation end products (AGEs), damages articular chondrocytes, and accelerates joint degeneration [93]. Furthermore, a high-sugar diet may reduce bone density, increase the risk of osteoporosis, and affect the stability of joints [94]. Moreover, a high-sugar diet leads to weight gain and increased joint load and may result in aggravated arthritis symptoms [95].

#### 3.1.3. High-Salt Diet

Sodium is the main component of edible salt, and it plays an essential role in the physiological function of mammals by maintaining the volume of extracellular fluid and the generation of cell membrane potential [96]. However, salt consumption may serve as an environmental risk factor for the development of autoimmune diseases. Moderate salt intake is necessary to maintain normal physiological function [97]. Excessive salt intake is associated with the risk of various non-communicable diseases, including hypertension, cardiovascular diseases, and stroke, which eventually leads to increased all-cause mortality [98].

A high-salt diet is also closely related to RA. A cross-sectional and case–control analysis of 18,555 people (including 392 patients with RA) revealed the relationship between dietary salt intake and RA. The median daily total sodium intake was 3.47 g, and the sodium intake increased the risk of RA in a dose-dependent manner [99]. Another nested case–control study was performed, and the results indicated that sodium intake significantly increased the risk of RA among smokers and even more than doubled the risk of RA for smokers [100]. The above studies indicated that a high-salt diet aggravated the risk of RA, and according to further studies, this effect of salt had generally been attributed to the activation of the inflammatory response. Recent studies had shown that increased sodium chloride intake contributes to the activation of pro-inflammatory macrophages (M1) and Th17 cells, significantly enhancing Th17 response, and reducing the number of T regulatory cells, which may increase the risk of RA [65]. In addition, a high-salt diet led to high salt concentration and hypertonicity, which directly activated serum glucocorticoid kinase (SGK1), thus affecting the sodium sensitivity and initial activation of Th17 cells, which might also contribute to RA development [66].

### 3.2. Balanced Diet Patterns

The balanced diet mode refers to a kind of diet that contains a variety of foods and aims to provide enough nutrients needed by the human body. This pattern emphasizes the diversity of foods and proper nutrient ratios to promote health and prevent chronic diseases [101]. The Mediterranean diet [102], the Dietary Approaches to Stop Hypertension (DASH) diet [103], and the vegetarian diet [104] are considered typical examples of balanced diets, among which the MD has generally been considered the healthiest diet for improving health and delaying aging.

The MD is a diet pattern based on the traditional diet mode in the Mediterranean area [105]. Its core characteristics include a high intake of vegetables, fruits, whole grains, nuts, olive oil, and beans [106]; a moderate intake of fish and poultry; and a low intake of dairy products, red meat, and sweets [107]. In addition, moderate consumption of red wine and seasoning intake of herbs and spices are encouraged [108]. This dietary pattern is rich in polyphenols, plant protein, healthy fat, and dietary fiber, which have multi-dimensional benefits for human health [109], including reducing the level of low-grade inflammation [38], improving cardiovascular health, lowering the risk of metabolic syndrome and diabetes [110], supporting cognitive function, and prolonging health.

(1) Anti-inflammatory effect: The MD is rich in antioxidants and anti-inflammatory components, such as polyphenols and omega-3 fatty acids, which can reduce the level of systemic inflammation [38,40]. In addition, the MD improved the symptoms of RA and lowered the level of inflammation. Studies have shown that the levels of inflammatory markers (such as CRP and IL-6) in the blood of RA patients with MD are reduced [111].
(2) Lipid profile improvement: Healthy fats in the MD (such as olive oil and fish oil) can improve the lipid profile by reducing the level of low-density lipoprotein (LDL) and increasing the level of high-density lipoprotein (HDL), thus lowering the risk of cardiovascular diseases and indirectly improving the health status of patients with RA [102].
(3) Immunoregulation: Some ingredients in the MD (such as prebiotics and probiotics) help to maintain gut microbiota balance and subsequently regulate immune system function, which may relieve the symptoms of RA [112]. The literature has shown that an imbalance in the gut microbiota is closely related to the occurrence of RA [113]. This imbalance may lead to abnormal reactions of the immune system, thus promoting inflammation. The prospect of probiotics as a potential intervention method is emphasized, and it is believed that by restoring the balance of the intestinal microflora, intestinal permeability could be improved, thus alleviating the symptoms of RA and regulating the immune response [113].
(4) Weight management: The MD is usually low in calories and high in fiber, which helps maintain a healthy weight and relieves the symptoms of RA [114].
(5) Intestinal health promotion: The MD is usually associated with an increase in intestinal microbial diversity. The Mediterranean diet is rich in fiber, which helps promote intestinal health and improve the diversity of the intestinal microflora, which may be related to the regulation of inflammatory reactions [115].

### 3.3. Mitigating Factors

Compared with an unbalanced diet, the MD is rich in whole grains, fruits, vegetables, and fermented foods (such as yogurt) [116], and it serves as an important source of dietary fiber, vitamins, and cellulose [117], which contributes to improved intestinal microbiota, enhanced immune system, and reduced inflammation [118] and may have a positive impact on RA patients.

#### 3.3.1. Dietary Fiber

Dietary fiber, which is widely found in foods such as vegetables, fruits, and whole grains, has been recognized as a very important functional food raw material and is called the seventh nutrient. Dietary fiber is usually divided into two types according to whether it is soluble in water: soluble dietary fiber (SDF) and insoluble dietary fiber (IDF). IDF has important swelling properties, whereas soluble fiber is fermented by some gut microbial flora to produce physiologically active metabolites.

Dietary fiber has been proven to be associated with various health benefits: (1) Altering the gut microbiota: Dietary fiber significantly enhances the diversity of intestinal microbiota, increases the relative abundance of beneficial bacteria, such as *Bifidobacterium* and *Lactobacillus*, and decreases the relative abundance of harmful bacteria, such as Gram-negative bacteria [119]. (2) Improving the intestinal barrier function: Dietary fiber stimulates intestinal epithelial cells to release mucus and forms a protective barrier resulting in improved natural defenses in the gut. In addition, dietary fiber also helps maintain the tight junction integrity of intestinal epithelial cells, resulting in reduced intestinal permeability and further contributing to an improved intestinal barrier [120]. (3) Production of beneficial metabolites: Secondary metabolites from beneficial bacteria in the gut, such as short-chain fatty acids (SCFAs), which can be significantly induced with the consumption of dietary fiber, provide various health benefits to the host, including regulating the immune system. SCFA is also closely related to the host’s metabolic activity, which plays a critical role in regulating blood sugar level, lipid metabolism, and energy balance [121]. (4) Weight management: Studies have shown that a high-fiber diet markedly increases satiety and decreased total calorie intake, thus contributing to improved weight management and a reduced risk of obesity [87].

Dietary fiber contributes to inhibiting the occurrence and development of RA. On the basis of data from the National Health and Nutrition Examination Survey (NHANES) from 2010 to 2020, high dietary fiber intake was closely associated with a significant reduction in the incidence of RA [44]. This effect of dietary fiber on RA could be at least partially attributed to the regulation of the immune system and inflammation. The results of another trial revealed that the number of circulating regulatory T cells significantly increased, the levels of markers of bone erosion dramatically decreased. And the prognosis of RA patients also improved in RA patients treated with high-fiber diets compared with those in the control group [67]. Further research showed that the increased levels of beneficial bacteria and their metabolite SCFAs were involved in this process. Arthritic mice that consumed resistant starch presented significant relief of arthritis symptoms, including swelling and pain, accompanied by an increased abundance of some beneficial bacteria and the production of propionic acid [68]. The results of a clinical trial with RA patients also revealed that an increase in dietary fiber led to a significant increase in systemic SCFA levels, especially the concentrations of acetic acid, propionic acid and butyric acid, and the level of inflammatory markers decreased significantly, indicating that the inflammatory response was alleviated [122]. Further study proved the role of SCFAs in the management of RA, as the severity of arthritis dramatically decreased and the development of RA was inhibited in mice with RA injected with butyric acid (a SCFA that can be produced by microbial decomposition of dietary fiber) [123]. The mechanism of this study involves butyric acid increasing the level of 5-hydroxyindole-3-acetic acid, activating aromatic hydrocarbon receptors, and altering the direction of B cell differentiation, thus alleviating arthritis and inhibiting the development of RA [123]. These studies indicate that their condition may be improved by adjusting their diet and provide a potential non-drug intervention strategy for arthritis patients.

#### 3.3.2. Probiotics

Probiotics are living microorganisms, such as lactic acid bacteria (*Lactococcus lactis*, *Lactobacillus acidophilus*), *bifidobacteria*, and yeast [124], that mainly colonize the human intestines and provide health benefits [125]. Fermented foods, including yogurt and kimchi, are the main source of probiotics in the diet. They are usually classified as “functional microbes” because they play massive roles in maintaining health [126]. The intake of probiotics helps to change the composition of the gut microbiota by both increasing the relative abundance of beneficial bacteria and suppressing the colonization and growth of pathogenic bacteria, which not only contributes to improved intestinal barrier function and reduces the occurrence of diarrhea and constipation but also promotes the absorption of nutrients such as vitamin K and regulates metabolism [127,128,129]. Recent studies have revealed that there is a connection between the intestine and the brain. Probiotics may improve mental states, such as anxiety and depression, by affecting the gut microbiota [130]. Moreover, probiotics also have certain beneficial effects on the immune system by promoting the activity of immune cells and secreting antibodies, thus improving resistance to infection [131]. Probiotics have a positive effect on relieving chronic inflammatory diseases, such as obesity [132].

As shown in Table 2, probiotics have shown significant mitigation effects on the development of RA. In a randomized, double-blind and placebo-controlled clinical trial, the levels of pro-inflammatory cytokines (such as TNF-α and IL-6) were significantly reduced in patients with *Lactobacillus casei* supplementation, and the clinical symptoms (such as joint swelling and pain) of patients also improved compared with those in the control group, indicating that probiotics may improve the symptoms of RA by adjusting the composition of the intestinal flora, enhancing the immune function, and reducing systemic inflammatory response [69,133]. Animal studies revealed similar results: a rat model of RA induced by Freund’s adjuvant (AIA) with continuous administration of different effective doses of probiotics resulted in less pain and significantly decreased levels of cytokines, and the infiltration of inflammatory cells decreased [70,134,135]. These results suggest that probiotics may be used as effective adjuvant therapies for the management of inflammatory joint diseases.

#### 3.3.3. Vitamins

Vitamins are essential nutrients needed by the human body and are widely present in foods, especially whole grains, nuts, green leafy vegetables, meat, and dairy products. Vitamin D is very important for the metabolism of calcium and phosphorus, which contributes to the health of bones, whereas vitamin D deficiency may lead to osteoporosis or rickets [136]. In addition, vitamin D assists in regulating the immune system and reducing inflammatory reactions. In vitro studies have shown that the active form of vitamin D [1,25-dihydroxyvitamin D, 1,25(OH)2D] has strong anti-proliferative, antibacterial, and anti-inflammatory properties [137].

It has widely been reported that a vitamin D deficiency is very common in patients with RA [138], and the level of serum vitamin D is negatively correlated with the incidence and disease activity of RA [139]. Thus, the level of serum 25-hydroxyvitamin D could be used as an index to evaluate the severity of RA. Furthermore, vitamin D supplementation significantly relieves joint pain, swelling, and dysfunction, and the levels of inflammatory markers such as CRP and TNF-α are obviously decreased [71]. These results indicate that vitamin D supplementation may be related to improvements of patients’ quality of life, especially in physical function and pain management.

Vitamin K plays an important role in blood coagulation and bone health, and a lack of vitamin K may lead to bleeding [140]. In addition, the effects of vitamin K2 [menadione-4 (MK-4)] on the proliferation of rheumatoid synovial cells and the development of arthritis induced by collagen (CIA) were studied, and the results showed that MK-4 inhibited the proliferation of FLSs and the occurrence of CIA in a dose-dependent manner [139]. Furthermore, the toxicity of vitamin K2 is obviously lower than that of other antiproliferative drugs, such as methotrexate (MTX) [141]. Vitamin K also plays an important role in bone health and mineralization, which may support the joint health of RA patients by affecting bone metabolism and reducing arthritis-related bone loss [72]. Therefore, vitamin K2 may constitute a new future strategy for the management of patients with RA.

Other vitamins may also indirectly contribute to the management of RA. For example, vitamin C participates in the synthesis of collagen, which helps to maintain the health of joint tissue [142]. Both vitamin C and vitamin E serve as antioxidants and may reduce the generation of free radicals in joints and further inhibit inflammation and joint injury [143].

#### 3.3.4. Minerals

Minerals such as calcium and phosphorus are closely related with bone health. Reports indicated that minerals level and oxidation stress were also involved in mitigating the symptom of RA [144,145]. Selenium, a recently attractive mineral component, exerts strong antioxidant effects. It is an important composition of selenoproteins, such as glutathione peroxidase (GPx), and contributes to suppressing the inflammatory reaction of RA by inhibiting the nuclear factor-kappa B (NF-κB) cascade and reducing the production of inflammatory mediators [146]. Studies showed that selenium nanoparticles supplementation improved the levels of tissue antioxidant enzymes and exerted anti-inflammatory effects by inactivating Adenosine 5‘-monophosphate (AMP)-activated protein kinase α (AMPKα)/mammalian target of rapamycin (mTOR) signal pathway [147,148]. In general, selenium supplementation contributed to reducing progression and symptoms of RA through suppressing oxidative stress level.

#### 3.3.5. Other Factor

Some dietary ingredients, such as omega-3 polyunsaturated fatty acids, are also involved in RA prevention. Reports have shown that a diet rich in omega-3 fatty acids is responsible for reducing inflammatory reactions and lowering RA-induced pain [149,150]. Supplementation with omega-3 fatty acids contributed to a decrease in the number of tender and swollen joints [39]. However, the effects of omega-3 fatty acids on preventing RA are still controversial. A meta-analysis revealed no beneficial effect of omega-3 fatty acid consumption on decreasing the risk of RA occurrence [151]. There is a need for longer durations and well-designed studies to further evaluate the influence of omega-3 fatty acid consumption on RA occurrence.

## 4. Conclusions

This review summarizes the pathogenesis of rheumatoid arthritis and the influence of different dietary patterns on RA. RA is a complex autoimmune disease, and its pathogenesis involves an inflammatory response and immune system disorders. Increasing evidence shows that a high-fat, high-sugar, and high-salt diet may promote the progression of RA, whereas a Mediterranean diet is considered to have a positive effect on RA. In addition, food ingredients, such as dietary fiber, probiotics, and vitamins, potentially reduce inflammation and relieve joint pain, which may play a key role in the treatment of RA. Although dietary modification may improve the quality of life of patients, the available evidence does not prove that dietary intervention can replace pharmacological treatment for RA.

Although detailed studies of the relationships between human dietary intake and immune and inflammatory pathways in RA are lacking, different underlying mechanisms have been proposed in vitro or in animal models. Therefore, diet may become an adjuvant therapy for standard RA treatment in the future, and it is suggested that patients with rheumatoid arthritis adopt a healthy lifestyle and nutritional strategy. Moreover, we should continue to explore the role and mechanism of the effects of diet on RA in the future and provide reliable guidance for the diet management of individuals with RA.

## Figures and Tables

**Table 1:** Diet of patients with RA and its effect on disease activity.

Diet Patterns	Dietary Types	Influencing Mechanism	References
Unbalanced diet patterns	High-fat diet	Long-term high-fat diet may lead to endocrine disorders, metabolic disorders, systemic low-grade chronic inflammation, reduce microbial diversity, and the integrity of the intestinal barrier, thus leading to the induction or aggravation of RA.	[61,62]
	High-sugar diet	High glucose intake aggravates RA symptoms by increasing blood glucose and insulin levels, increasing inflammatory factors, accelerating joint degeneration, reducing bone mineral density, and leading to weight gain.	[63,64]
	High-salt diet	Increasing the intake of sodium chloride may decrease the number of T regulatory cells by activating pro-inflammatory macrophages and Th 17 cells and affecting the sodium sensitivity of Th 17 cells, thus increasing the risk of RA.	[65,66]
Balanced diet patterns	Dietary fiber-rich diet	Dietary fiber may help alleviate the symptoms of rheumatoid arthritis by regulating inflammation, improving intestinal microbial community, controlling body weight, enhancing immune function, and maintaining intestinal barrier.	[67,68]
	Probiotics-rich diet	Probiotics can significantly alleviate the occurrence and development of rheumatoid arthritis through immunomodulation, inhibiting inflammation, maintaining intestinal microbial balance, enhancing intestinal barrier function, and improving nutrient absorption.	[69,70]
	Vitamins-rich diet	Vitamins may play an active role in the management and treatment of rheumatoid arthritis through anti-inflammatory reaction, immunomodulation, antioxidation, and promoting bone health.	[71,72]

**Table 2:** Study of the therapeutic effect of probiotics in RA.

Study Size and Duration	Probiotic Strain	Results	References
Forty-six patients with RA (8 weeks)	*Lactobacillus casei*	Disease activity score was significantly decreased by the intervention, a statistically significant improvement in all pro-inflammatory biomarkers except for IL-1β.	[69]
Sixty patients with RA (8 weeks)	*Lactobacillus acidophilus* *Lactobacillus casei* *Bifidobacterium bifidum*	Taking probiotic supplements for 8 weeks among patients with RA had beneficial effects on DAS-28, insulin levels, HOMA-B, and hs-CRP levels.	[133]
Male DBA/1J mouse model of RA (7 weeks)	*L. helveticus* SBT2171	The ability of *L. helveticus* SBT2171 to downregulate the abundance of immune cells and the subsequent production of CII-specific antibodies and IL-6, thereby suppressing the CIA symptoms.	[134]
Male Wistar rat model of RA (4 weeks)	*Lactobacillus casei* and *Lactobacillus acidophilus*	Treatment with *Lactobacillus casei* and *Lactobacillus acidophilus* significantly downregulated pro-inflammatory cytokines and upregulated anti-inflammatory cytokines and significantly reduced the oxidative stress and arthritis score of synovial tissues.	[135]

## Abbreviations

The following abbreviations are used in this manuscript:

RA	Rheumatoid arthritis
MD	Mediterranean diet
HLA	Human leucocyte antigen
PTPN22	Protein tyrosine phosphatase, non-receptor type 22
CTLA-4	Cytotoxic T lymphocyte-associated antigen-4
CRP	C-reaction protein
IL	Interleukin
TNF	Tumor necrosis factor
MMP	Metalloproteinase
AGEs	Advanced glycation end products
SGK1	Serum glucocorticoid kinase
DASH	Dietary Approaches to Stop Hypertension
LDL	Low-density lipoprotein
HDL	High-density lipoprotein
IDF	Insoluble dietary fiber
SDF	Soluble dietary fiber
SCFAs	Short-chain fatty acids
MTX	Methotrexate
MK-4	Menadione-4
CIA	Arthritis induced by collagen
AMPK	Adenosine 5‘-monophosphate (AMP)-activated protein kinase
mTOR	Mammalian target of rapamycin
